# The Precautionary Principle and Risk Perception: Experimental Studies in the EMF Area

**DOI:** 10.1289/ehp.7538

**Published:** 2005-01-10

**Authors:** Peter M. Wiedemann, Holger Schütz

**Affiliations:** Research Centre Jülich, Programme Group MUT (Humans, Environment, Technology), Jülich, Germany

**Keywords:** base stations, electromagnetic fields, health protection, mobile phones, precautionary principle, risk perception

## Abstract

Possible adverse health effects due to electromagnetic fields (EMFs) from cellular phones and base stations present a major public health issue across Europe. Because scientists cannot exclude that EMFs may cause health problems, the application of the precautionary principle is debated heavily. By considering precautionary measures, political decision makers hope to cope with public fears about EMFs. We present results from two experimental studies that indicate that precautionary measures may trigger concerns, amplify EMF-related risk perceptions, and lower trust in public health protection. Such impacts, questioning common expectations, should be considered in decisions about precautionary measures.

The public debate about possible adverse health effects from exposure to electromagnetic fields (EMFs) from cellular phones and base stations is one of the risk issues that occupies many political decision makers across Europe (Burgess 2004). Because scientists cannot exclude the possibility that EMFs may cause health problems [Independent Expert Group on Mobile Phones (IEGMP) 2000; National Radiological Protection Board (NRPB) 2003; Strahlenschutzkommission (SSK) 2001], the application of the precautionary principle is heatedly discussed in many countries. For instance, the IEGMP indicated that the balance of evidence showed no adverse health effects from exposure to radio frequency radiation from mobile phone technologies. However, the group still recommended that “a precautionary approach to the use of mobile phone technologies be adopted until much more detailed and scientifically robust information on any health effects becomes available” (IEGMP 2000, p. 3).

Essentially, the precautionary principle recommends that action should be taken to prevent serious potential harm, regardless of scientific uncertainty as to the likelihood, magnitude, or cause of that harm. By considering precautionary measures, political decision makers hope to cope with these public fears about EMFs. Various courses of action are taken into consideration, including health-related measures such as exposure minimization strategies or stricter exposure limits, process-related measures such as better risk communication and enhancing public participation in base station siting decisions, and research-related measures (Wiedemann et al. 2001). In various countries, different options have been chosen, such as participatory site selection of base stations in the Netherlands, stricter exposure limits in Switzerland, and better risk communication in the United Kingdom (public access to databases revealing the sites and technical features of the base stations), as well as labeling of cellular phones (discussed also in Germany) and general exposure reduction measures, just to name a few [Bundesamt für Strahlenschutz (BfS) 2004; NRPB 2005; TCO 2001].

Although the theoretical status and rationality of the precautionary principle have been discussed in many papers (Commission of the European Communities 2000; Foster et al. 2000; Kriebel et al. 2001; Marchant 2003) and conferences [Grandjean et al. 2003; Raffensberger and Tickner 1999; World Health Organization (WHO) 2003], only a few empirical studies analyze the impact of precautionary measures on risk-related attitudes and beliefs.

## Risk Perceptions as Triggers for Precautionary Action

Whether public risk perception should be a stimulus for invoking precautionary measures in risk management is a sensitive question (Goldstein and Carruth 2004). Opponents to this approach stress the point that risk management should be based on sound science using the best available scientific evidence. They assume that perceived risk differs from assessed risk in that it may more readily be manipulated. In addition, they fear that precautionary measures may undermine the scientific basis for the established exposure limits. In their view, precautionary measures for EMFs should be adopted only with great care.

Proponents argue that public risk perception should be taken into account in decisions about risk management: When the public is concerned about a risk, risk managers should address these concerns by invoking additional protective measures. Furthermore, they underline that societal values and public willingness to accept a risk are key factors in determining a society’s level of protection. Thus, public risk perception must be recognized as a factor in the decision to apply precautionary measures. That is, in addition to scientific data, knowledge gained from the practical experience of professionals and risk perceptions of lay people are seen as a valid basis for making decisions about when to invoke precautionary measures (e.g., Gee and Stirling 2003; Tickner 2003).

## Research Questions

Several studies have investigated the impact of risk communication on risk perception (e.g., MacGregor et al. 1994; Morgan et al. 1985; Purchase and Slovic 1999; Schütz and Wiedemann 1995). However, to date, no one—at least to our knowledge—has addressed empirically the question of whether the communication of precautionary measures influences risk perceptions and, if so, in which direction. This is astonishing, especially because risk perceptions play a prevalent role in the discussion about the necessity of involving the precautionary principle.

In this article we focus on the issue of how people react to the implementation of the pre-cautionary principle. The key is the impact of precautionary measures on risk perceptions. Two opposing hypotheses can be derived from the current available literature. First, pre-cautionary measures will increase trust in risk management, and, in turn, increased trust in risk management will be associated with lower risk perceptions. Second, the alternative hypothesis points to the possibility that pre-cautionary measures will be considered a cue that the risk might be real. Here, perceived risk should be amplified.

As discussed above, the reason for invoking the precautionary principle is scientific uncertainty. Thus, it would be of interest to see whether emphasizing the uncertainty in scientific knowledge about EMF risks will affect risk perception. We conducted two experiments to address these questions. In the first experiment, health-related precautionary measures served as stimuli; in the second experiment, a process-related precautionary measure was used: public participation.

## Experiment 1

The first experiment focused on the effect of two independent variables: *a*) health-related precautionary measures and *b*) experts’ uncertainty about the sufficiency of health protection. Two dependent variables were used: perceived risk of electrosmog and the perceived quality of scientific knowledge about health risks from electrosmog.

### Materials and methods.

These questions were investigated in an experimental study using 4 × 2 factorial design. The first factor was composed of a basic text and three different precautionary measures (see Table 1). In the “no precaution” condition, only the basic text was presented. In the three “precaution” conditions, the basic text and one of the descriptions of precautionary measures were provided. Those descriptions used phrases that reflect measures and arguments actually used in regulating the siting of base stations in Germany.

The second factor varied the emphasis of uncertainty. In the “uncertainty” condition, a sentence that pointed to scientific uncertainty about the sufficiency of current protection measures was included in the basic text. In the “no uncertainty” condition, this sentence was missing (see Table 1).

An Austrian ad hoc sample of 246 subjects 18–81 years of age, with a median age of 24 years (62% female, 38% male), answered a questionnaire that included one of the eight texts from the experimental conditions. Sampling occurred in October 2003 among students and employees of the University of Innsbruck, and subjects were randomly assigned to the experimental conditions. Risk perceptions and perceived quality of scientific knowledge were collected with a 7-point rating scale asking, “All in all, how threatened do you feel about electrosmog?” (1 = “I don’t feel threatened at all”; 7 = “I feel very threatened”) and, “How do you rate the scientific knowledge about the health risks of electrosmog?” (1 = “In science the knowledge is quite deficient”; 7 = “Scientific knowledge is quite good”). Subjects were explicitly instructed to answer the questions from their own subjective perspective, that is, referring to their beliefs.

At the beginning of the questionnaire, all participants were asked to indicate their risk perceptions for the following items (on 7-point rating scales): bovine spongiform encephalopathy (BSE), nuclear power, smoking, genetically engineered foodstuffs, climate change, and crime. Because these risk judgments were made before the introduction of the experimental manipulations, they can serve as an additional check whether—despite the random assignment of the subjects to the experimental conditions—there were any differences in risk perceptions among the experimental groups that might confound the results of this experiment.

### Results.

**Figure 1:**
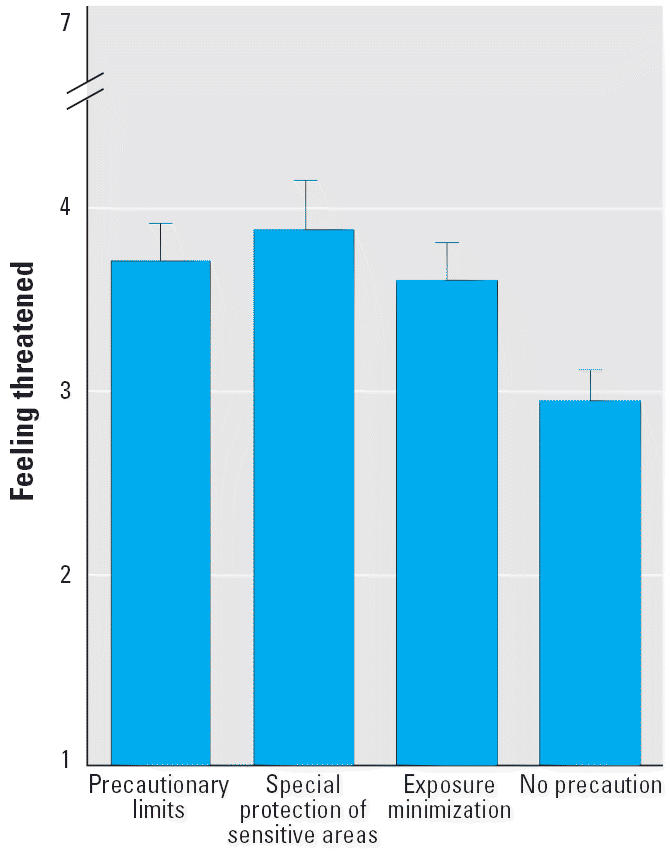
Mean ratings (± SEM) for the four “precautionary measures” conditions.

For risk perception, a two-way analysis of variance (ANOVA) yielded a statistically significant main effect for the pre-cautionary measures factor (*F*_3,238_ = 3.954; *p* = 0.009) and a statistically insignificant main effect for the uncertainty factor (*F*_1,238_ = 0.730; *p* = 0.394). There was no statistically significant interaction between the two factors (*F*_3,238_ = 0.343; *p* = 0.794). Figure 1 shows the average ratings for each of the four conditions of the precautionary measures factor. Clearly, the mean for the “no precaution” condition is much lower than the means for the three “precautionary measures,” which in turn are all close together.

A separate analysis by means of a post hoc test (Tukey HSD) confirms this visual impression. It is the “no precaution” condition that is statistically different (*p* < 0.05) from “special protection of sensitive areas” and “precautionary limits,” and marginally statistically different (*p* = 0.074) from “exposure minimization.” The three “precautionary measures” conditions do not differ significantly from each other.

To determine whether these significant effects were produced by experimental variation, we conducted separate ANOVAs between the eight experimental treatment groups (resulting from the two factors “precautionary measures” and “uncertainty”) for the other risk items appraised before the experimental variation (BSE, nuclear power, smoking, genetically engineered foodstuffs, climate change, and crime) as dependent variables. None of these six ANOVAs yielded a statistically significant effect. This supports the notion that it was in fact the experimental manipulation that produced the differences in risk perception, and not some chance effect.

For the second dependent variable, the perceived quality of scientific knowledge about potential health risks of electrosmog, we found no statistically significant effect.

## Experiment 2

The second experiment focused on the impact of a process-related precautionary measure on perceived risk of electrosmog, perceived quality of scientific knowledge and—as an additional variable—trust in public health protection. As in the first experiment, we also varied the experts’ uncertainty about the sufficiency of health protection.

### Materials and methods.

In this experiment we used a 2 × 2 factorial design. The first factor was composed of a basic text (identical to the one given in the first experiment) and the “public participation” precautionary measure (Table 2). In the “no precaution” condition, only the basic text was presented. In the “precaution” conditions, the basic text plus the text about the public precaution measure was provided. The second factor was identical to the one used in the first experiment: In the “uncertainty” condition, a sentence that pointed to scientific uncertainty about the sufficiency of current protection measures was included in the basic text. In the “no uncertainty” condition, this sentence was missing (Table 2).

Three 7-point rating scales were used to collect the ratings for the dependent variables (risk perception, trust in health protection, and quality of scientific knowledge). The wording of the scales was as follows: Risk assessment: “All in all, how threatened do you feel about electrosmog?” (1 = “I don’t feel threatened at all”; 7 = “I feel very threatened”); trust: “How much do you trust that the health protection of the public is ensured?” (1 = “no at all”; 7 = “completely”); state of the scientific knowledge: “How do you rate the knowledge about the health effects of electrosmog?” (1 = “the knowledge is quite deficient”; 7 = “the knowledge is quite good”).

Eighty-four Austrian subjects, recruited in March 2004 among students and employees of the University of Innsbruck, participated in this experiment. Subjects were randomly assigned to one of the four experimental conditions (19–45 years of age; median age, 23 years; 76% female, 24% male). Each subject received a sheet showing the respective text of the experimental condition and the three response scales on risk perception, scientific knowledge, and trust. Subjects were asked to read the text and then to give their ratings on the three scales. Again, subjects were explicitly instructed to answer the questions from their own subjective perspective, that is, referring to their beliefs.

### Results.

For each of the three dependent variables, we conducted a separate two-way ANOVA. For both “risk perception” and “perceived quality of scientific knowledge,” we found no statistically significant main effect for the precautionary measures factor. However, for “trust in health protection,” the ANOVA yielded a statistically significant main effect for the precautionary measure factor (*F*_1,80_ = 5.533; *p* = 0.021).

**Figure 2:**
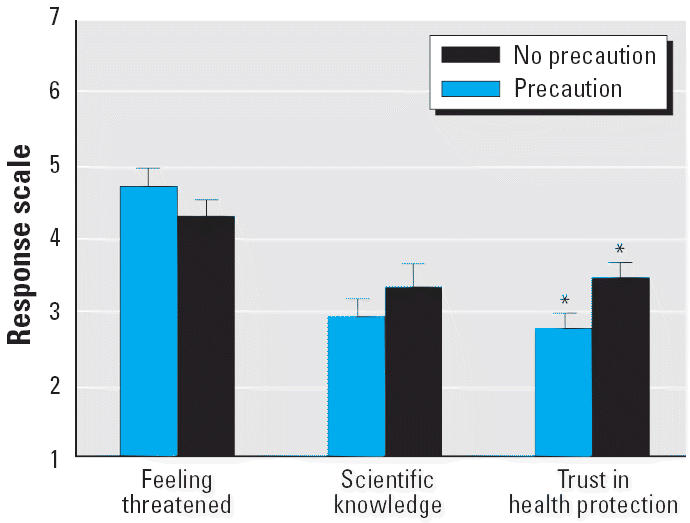
Mean ratings (± SEM) for the two “precautionary measures” conditions for each of the three dependent variables.
*p < 0.05.

Figure 2 shows, for each of the three dependent variables, the average ratings for the two conditions of the precautionary measures factor. For trust, the ratings were lower in the precaution condition. As in the first experiment, there was no statistically significant main effect of the “uncertainty” factor for any of the dependent variables.

## Discussion

The results of our first experiment strongly support the second hypothesis stated above: that precautionary measures will be considered a cue that a risk might be real and increase perceived risk. In experiment 1, the mean responses for “feeling threatened” were higher in the three “precaution” conditions than in the “no precaution” condition. Note that also in the second experiment (using the “public participation” as the precautionary measure), the results are in the same direction: Under the “precaution” condition, the mean ratings for “feeling threatened” were higher than under the “no precaution” condition—however, the difference did not reach statistical significance.

The second experiment indicates that “public participation” precautionary measures do not increase trust in public health protection. This result speaks against the first hypothesis, which states that precautionary measures will increase trust in risk management, and, in turn, that increased trust in risk management will be associated with lower risk perceptions.

One may argue that, although statistically significant, the reported effects are small and thus may not be of practical relevance. But no matter how small the effects are, they are contrary to the expectations of policy makers who hope to calm public fears about EMFs by implementing precautionary measures.

The second variable manipulated in the two experiments was the scientific uncertainty about the sufficiency of current protection measures. This manipulation did not affect any of the dependent variables (perceived risk, scientific knowledge, trust in public health protection). This is surprising because it is this uncertainty that actually provides the reason for applying the precautionary principle. So one would have expected an effect—at least for the “scientific knowledge” variable. One can only speculate why this was the case. Perhaps the experimental manipulation was simply not strong enough.

## Conclusions

Precautionary measures implemented with the intention of reassuring the public about EMF risk potentials seem to produce the opposite effect. They may amplify EMF-related risk perceptions and trigger concerns. Referring to the WHO definition of health [“a state of complete physical, mental and social well-being and not merely the absence of disease or infirmity” (WHO 1948)], it seems that precautionary measures themselves can be precarious because they might impair well-being.

The results of the two experiments support the warnings in the WHO background document (WHO 2000) on cautionary policies “that such policies be adopted only under the condition that scientific assessments of risk and science-based exposure limits should not be undermined by the adoption of arbitrary cautionary approaches.” We tend to add that any precautionary policy should consider possible countervailing risks such as increasing fear and unnecessarily spreading anxieties. These adverse impacts of precaution should be brought to the attention of policy makers.

Of course, these results need to be confirmed in further experiments before drawing practical conclusions for cautionary policies. They also pose a number of questions for further research. For instance, why did the uncertainty condition (i.e., the reference to scientific uncertainty about the sufficiency of current protection measures) have no effect on risk perception, trust, or scientific knowledge? And even more important, are there any conditions under which application of precautionary measures will increase trust in risk management, which in turn will result in lower risk perceptions?

## Figures and Tables

**Table 1:** Text fragments used in experiment 1.

Experimental condition	Text
Basic text	A widespread debate about the potential risks related to electrosmog is ongoing. Some scientists argue that substantial uncertainties exist as to whether current protection from electrosmog is sufficient.*^a^*
	The International Commission for (Nonionizing) Radiation Protection points out that current exposure limits protect the public adequately.
Minimization	Nevertheless, the commission recommends precautionary measures: exposure from mobile phone emission is to be kept as low as possible.
Special protection of sensitive areas	Nevertheless, following a precautionary approach, many local communities demand that base stations should not be sited near sensitive locations such as day care facilities, schools, or hospitals.
Precautionary limits	Following a precautionary approach, Switzerland has tightened exposure limits by a factor of 10 in areas where people are exposed for long periods of time.

a Sentence added in the “uncertainty” condition of the second experimental factor.

**Table 2:** Text fragments used in experiment 2.

Experimental condition	Text
Basic text	Presently there is widespread debate about the potential risks related to electrosmog. Some scientists argue that substantial uncertainties exist as to whether current protection from electrosmog is sufficient.*^a^*
	The International Commission for (Nonionizing) Radiation Protection points out that current exposure limits protect the public adequately.
Public participation	However, for precautionary reasons, many local authorities claim that the residents should be involved in the siting process for base stations.

a Sentence added in the “uncertainty” condition of the second experimental factor.
